# Mutations at Beta N265 in γ-Aminobutyric Acid Type A Receptors Alter Both Binding Affinity and Efficacy of Potent Anesthetics

**DOI:** 10.1371/journal.pone.0111470

**Published:** 2014-10-27

**Authors:** Deirdre S. Stewart, David W. Pierce, Mayo Hotta, Alex T. Stern, Stuart A. Forman

**Affiliations:** Department of Anesthesia Critical Care and Pain Medicine, Massachusetts General Hospital, Boston, Massachusetts, United States of America; Indiana University School of Medicine, United States of America

## Abstract

Etomidate and propofol are potent general anesthetics that act via GABAA receptor allosteric co-agonist sites located at transmembrane β+/α− inter-subunit interfaces. Early experiments in heteromeric receptors identified βN265 (M2-15′) on β2 and β3 subunits as an important determinant of sensitivity to these drugs. Mechanistic analyses suggest that substitution with serine, the β1 residue at this position, primarily reduces etomidate efficacy, while mutation to methionine eliminates etomidate sensitivity and might prevent drug binding. However, the βN265 residue has not been photolabeled with analogs of either etomidate or propofol. Furthermore, substituted cysteine modification studies find no propofol protection at this locus, while etomidate protection has not been tested. Thus, evidence of contact between βN265 and potent anesthetics is lacking and it remains uncertain how mutations alter drug sensitivity. In the current study, we first applied heterologous α1β2N265Cγ2L receptor expression in Xenopus oocytes, thiol-specific aqueous probe modification, and voltage-clamp electrophysiology to test whether etomidate inhibits probe reactions at the β-265 sidechain. Using up to 300 µM etomidate, we found both an absence of etomidate effects on α1β2N265Cγ2L receptor activity and no inhibition of thiol modification. To gain further insight into anesthetic insensitive βN265M mutants, we applied indirect structure-function strategies, exploiting second mutations in α1β2/3γ2L GABAA receptors. Using α1M236C as a modifiable and anesthetic-protectable site occupancy reporter in β+/α− interfaces, we found that βN265M reduced apparent anesthetic affinity for receptors in both resting and GABA-activated states. βN265M also impaired the transduction of gating effects associated with α1M236W, a mutation that mimics β+/α− anesthetic site occupancy. Our results show that βN265M mutations dramatically reduce the efficacy/transduction of anesthetics bound in β+/α− sites, and also significantly reduce anesthetic affinity for resting state receptors. These findings are consistent with a role for βN265 in anesthetic binding within the β+/α− transmembrane sites.

## Introduction

**Figure 1:**
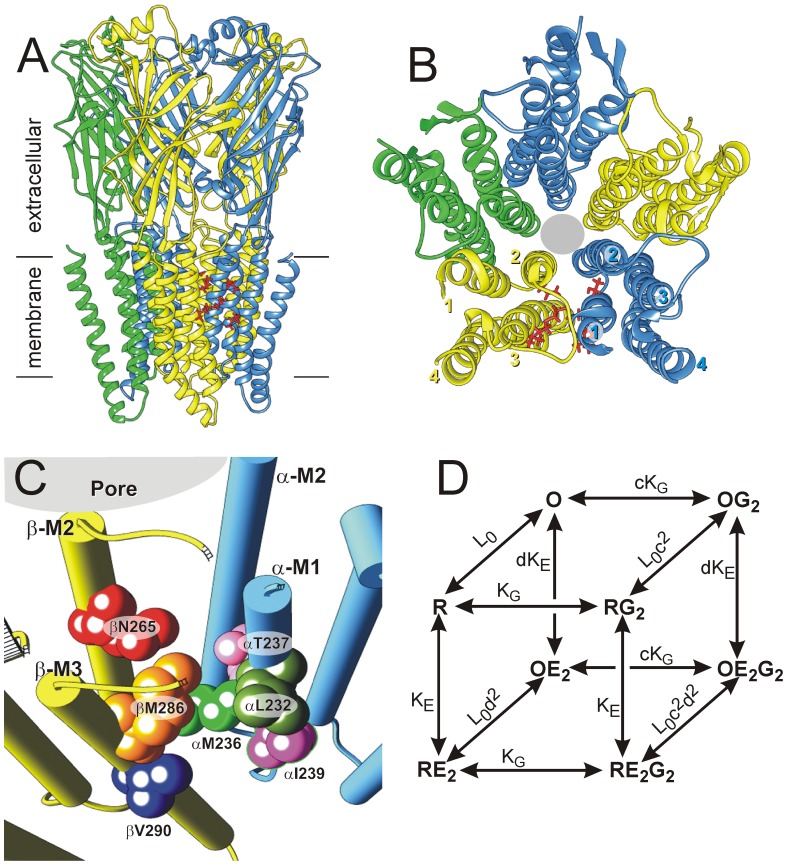
Functional and structural models of GABA_A_ receptor interactions with etomidate. **Panel A:** A side-on ribbon depiction of a structural homology model for α1β3γ2L GABA_A_ receptors based on the glutamate-gated chloride channel (GluCl) from *Caenorhabditis elegans* [20]. Both the extracellular domains and transmembrane domains are shown in relation to membrane lipids. Subunits are color-coded (α1 = blue; β3 = yellow; γ2L = green). βN265 and other residues involved in etomidate and propofol binding are depicted as red stick structures in one of two β+/α− transmembrane interfacial sites. **Panel B:** A view of the transmembrane domains from the extracellular space shows the structure of each subunit’s four-helix bundle and the arrangement of subunits around the central chloride channel (grey circle). Residues in one interfacial anesthetic site are depicted as red stick structures. **Panel C:** A close-up view of one β+/α− transmembrane inter-subunit etomidate binding site in the homology model. Helix backbones are depicted as solid cylinders. The βN265 residue and six anesthetic contact residues identified by photolabeling or cysteine modification/protection are highlighted as labeled space-filling structures. **Panel D:** A Monod-Wyman-Changeux two-state (inactive = R; active = O) equilibrium co-agonist scheme with two equivalent orthosteric agonist (GABA; G) sites and two equivalent allosteric agonist (etomidate; E) sites is depicted [1]. For simplicity, states with only one occupied agonist site are omitted. The model is defined by five equilibrium parameters (see Eq. 3, methods): L_0_ is a basal gating equilibrium (C/O); K_G_ and K_E_ are dissociation constants for respectively, GABA and etomidate binding to inactive receptors; c and d quantify the binding affinity ratios for respectively, GABA and etomidate to active vs. inactive receptors. Maximal agonist efficacies for GABA and etomidate are respectively, (1+L_0_c^2^)^−1^ and (1+L_0_d^2^)^−1^.

Etomidate and propofol are potent general anesthetics that act as positive allosteric modulators and agonists at GABA_A_ receptors [1]–[3], the major inhibitory neurotransmitter receptors of the central nervous system and members of the pentameric ligand-gated ion channel (pLGIC) superfamily [4], [5]. Typical synaptic GABA_A_ receptors consist of α, β, and γ subunits, arranged β-α-β-α-γ counterclockwise viewed from the extracellular space [6]. Structural homology models of αβγ GABA_A_ receptors, based on crystallography of distantly related pLGICs [5] and β3 homo-pentameric channels [7], are consistent with a large body of structure-function data. Each homologous subunit has a large extracellular domain, and four α-helices (M1 to M4) forming a transmembrane domain (Fig. 1A, B). The two GABA binding sites are located in the β+/α− interfaces of the extracellular domain. The transmembrane domains of each subunit form four-helix bundles with all five M2 domains around the central chloride channel (Fig. 1B). Surrounding the M2 domains is a second ring of M1 and M3 α-helices, with M4 domains outermost. Portions of M1, M3 and M4 contact membrane lipids.

Allosteric co-agonist sites for etomidate and propofol are located between the extracellular ends of α-M1 and β-M3 transmembrane helices; the transmembrane β+/α− interfaces (Fig. 1B, C). Combined, photolabeling with etomidate and propofol analogs [8]–[10], photolabel inhibition studies [10], [11], and substituted cysteine accessibility method (SCAM) with protection experiments [12]–[14] have identified six residues near either etomidate or propofol: βM286 and βV290 on β-M3 as well as αL232, αM236, αT237, and αI239 on α-M1 (Fig. 1C). Tryptophan mutations at either αM236 or βM286, two photolabeled residues, mimic the channel gating effects of bound anesthetics and reduce sensitivity to both etomidate and propofol [15], [16]. Disulfide bond formation between engineered cysteines demonstrates that the photolabeled faces of α-M1 and β-M3 abut water-accessible transmembrane clefts [17], [18]. Additionally, high resolution crystallography identifies homologous inter-subunit transmembrane pockets where ivermectin, an allosteric modulator/agonist of various pLGICs [19], binds to *Caenorhabditis elegans* glutamate-sensitive chloride channels (GluCl) [20].

In addition to the α-M1 and β-M3 residues identified above, M2 domain residues may contribute to the β+/α− transmembrane sites for propofol and etomidate. The focus of this study, β-M2 position 265 (M2-15′), is of particular and longstanding interest as one of the first sites where mutations were found to affect GABA_A_ receptor sensitivity to alcohols and anesthetics [21], [22]. This residue also plays a critical role in determining etomidate and propofol sensitivity in mammalian GABA_A_ receptors. Interchanging the β-M2-15′ asparagine of β2 or β3 and the homologous serine of β1 accounts for the remarkable specificity of etomidate for receptors containing β2/3 subunits [22], [23]. Methionine substitution at β2 or β3 N265 eliminates etomidate sensitivity and weakens propofol effects at the receptor level [24], [25], and β3N265M transgenic mice show remarkable resistance to both anesthetics [26]. Quantitative mechanistic comparison of etomidate effects in voltage-clamped wild-type α1β2γ2L and α1βN265Sγ2L receptors expressed in *Xenopus* oocytes indicated that drug efficacy was reduced and that affinity for receptors might be weakened. Similar studies of α1βN265Mγ2L receptors were inconclusive due to the absence of anesthetic effects [27]. Homology models of αβγ GABA_A_ receptors locate βN265 either near or within the β+/α− site (Fig. 1C), and *in silico* docking calculations suggest possible contact with anesthetics [9], [12], [28], [29]. Moreover, contact with M2-15′ residues is evident in x-ray diffraction structures demonstrating allosteric modulators bound within inter-subunit sites of crystallized pLGIC homologs: ivermectin contacts M2-15′ of GluCl [20], while ethanol and the alkane anesthetic bromoform contact M2-15′ sidechains in positively modulated prokaryotic *Gloeobacter violaceus* ion channel (GLIC) mutants [30]. Nonetheless, no anesthetic photolabel incorporation at GABA_A_ βN265 has been detected. One photoreactive propofol analog incorporates at β3H267 [31], which is predicted to be on the M2 helix face opposite the β+/α− interface. A SCAM protection study of α1βN265Cγ2L receptors reported no propofol protection [13], while another reported protection by n-octanol [32]. No studies have reported whether etomidate protects βN265C from modification. Thus, it remains uncertain whether βN265 mutations impair only the efficacy of etomidate and propofol, a result suggesting indirect interactions, or also the affinity of these drugs for GABA_A_ receptors, consistent with a role in binding.

To discriminate between the effects of βN265 mutations on anesthetic binding *versus* efficacy, we applied both direct and indirect structure-function approaches. We first performed pharmacological sensitivity and thiol modification-protection studies of α1β2N265Cγ2L GABA_A_ receptors expressed in *Xenopus* oocytes and monitored with voltage-clamp electrophysiology, using etomidate at concentrations up to 300 µM (intending to achieve high site occupancy). These experiments revealed that etomidate neither affects α1β2N265Cγ2L receptor function nor inhibits reaction with a water-soluble thiol modifier. We also explored βN265M effects in GABA_A_ receptors containing previously characterized second point mutations: a modifiable and anesthetic-protectable cysteine (α1M236C), and a mutation that mimics bound anesthetic (α1M236W). Our results reveal that βN265M reduces both anesthetic binding affinity at β+/α− sites and transduction of gating effects associated with anesthetic binding.

## Methods

### Animal use

Adult female *Xenopus laevis* frogs were used as a source of oocytes for voltage-clamp electrophysiological experiments. Frogs were housed in a veterinarian-supervised facility and used in accordance with the NIH Guide for the Care and Use of Laboratory Animals. All animal procedures for this study were approved by the Massachusetts General Hospital Institutional Animal Care and Use Committee (Offices of Laboratory Animal Welfare assurance #A3596-01; MGH protocol 2005N000051). To ameliorate suffering, frogs were anesthetized by immersion in 0.2% tricaine prior to harvesting oocytes (Sigma-Aldrich, St. Louis, MO). Laparotomy wounds were minimized (<0.5 cm), and infiltrated with 0.25% bupivicaine to provide post-procedure analgesia. To reduce the number of frogs used, each frog was subjected to a maximum of six oocyte harvests with at least 8 weeks recovery between procedures.

### Chemicals

R(+)-Etomidate (2 mg/ml in 35% propylene glycol:water) was from Bedford Laboratories (Bedford, OH). Propofol was purchased from Sigma-Aldrich (St. Louis, MO) and stored as stock solution in DMSO. Alphaxalone was purchased from MP Biomedical (Solon, OH) and prepared as a stock solution in DMSO. Drugs were diluted into electrophysiology solutions on the day of use. Maximal experimental concentrations of propylene glycol (<4%) and DMSO (≤0.1%) produced no functional effects on GABA_A_ receptors [1]. Picrotoxin (PTX; from Sigma-Aldrich) was dissolved in electrophysiology buffer (2 mM). *p*-Chloromercuribenzenesulfonic acid sodium salt (pCMBS) was purchased from Toronto Research Chemicals (North York, Ontario, Canada). Salts and buffers were purchased from Sigma-Aldrich.

### Molecular Biology

cDNAs for human GABA_A_ receptor α1, β2, and γ2L subunits were cloned into pCDNA3.1 vectors (Invitrogen, Carlsbad, CA). Plasmids encoding concatenated dimer (β2-α1 and β3-α1) and trimer (γ2-β2-α1 and β3-α1-δ) subunit proteins were a generous gift from Professor Erwin Sigel (Institute for Biochemistry & Molecular Medicine, University of Bern, Switzerland) [6], [33], [34]. A β3-α1-γ2L trimer cDNA construct was created by excising δ and appending γ2L using overlap extension polymerase chain reaction. The β2-α1 (rat) constructs contain a 26 residue linker (Q_5_A_3_PAQ_2_A_3_PA_2_Q_5_) between the C-terminus of β2 and the N-terminus of the mature α1 (without its leader sequence). The γ2L-β2-α1 trimer also contains a 23 residue linker (Q_5_A_3_PAQ_3_AQA_3_PA_2_Q_5_) between γ2 and β2. In β3-α1 and β3-α1-γ2L constructs (rat β3, human α1 and γ2L), linkers between β3 and α1 were composed of 23 amino acids (Q_5_A_3_PTGQA_3_PA_2_Q_5_), and that between α1 and γ2L subunits is 10 amino acids (Q_4_TGQ_4_). Mutations in cDNAs were created with oligonucleotide-directed mutagenesis using QuikChange kits (Agilent Technologies, Santa Clara, CA). Clones from each mutagenesis reaction were subjected to DNA sequencing through the entire cDNA region to confirm the presence of the mutation and absence of stray mutations.

### Oocyte Electrophysiology

Messenger RNA synthesis and *Xenopus* oocyte expression were performed as previously described [16]. Electrophysiological experiments were done at 21 to 23°C. All drugs were delivered in ND96 electrophysiology buffer (in mM: 96 NaCl, 2 KCl, 0.8 MgCl_2_, 1.8 CaCl_2_, 5 HEPES, pH 7.5). Peak current responses to varying GABA concentrations (range 0.1 µM to 10 mM) alone or co-applied with anesthetics, were measured in *Xenopus* oocytes using two microelectrode voltage clamp electrophysiology, as previously described [12]. The duration of GABA application varied depending on the time to reach steady-state peak current. Responses to maximal GABA (1 to 10 mM), were recorded every 2^nd^ or 3^rd^ sweep for normalization. Picrotoxin-sensitive spontaneous channel activity was measured by applying 2 mM PTX, followed by >5 minute washout and a maximal GABA response test. Etomidate (10 µM) or alphaxalone (2 µM) were used as gating enhancers with high GABA concentrations to estimate maximal GABA efficacy. Oocyte currents were low-pass filtered at 1 kHz (Model OC-725B, Warner Instruments, Hamden, CT) digitized at 1–2 kHz (Digidata 1200, Molecular Devices, Sunnyvale, CA) and recorded digitally (pClamp 7, Molecular Devices).

### Cysteine Modification with pCMBS and Etomidate Protection in Xenopus Oocytes

The pCMBS concentrations used for modification experiments were chosen so that initial modification rate conditions (less than 50% of maximal effect) were maintained at up to 40 s exposure. Oocytes were repetitively stimulated with GABA pulses every five minutes until at least three sequential current responses were constant (±5%). For modification and protection experiments, oocytes were exposed to pCMBS (alone, with GABA, with anesthetic, or with GABA+anesthetic) for 5 or 10 s, followed by 5–10 min wash in ND96. Responses to both low GABA (EC10) and high GABA (1 to 3 mM) were tested after each cycle of pCMBS exposure and wash. After up to ten cycles of modification and wash, maximal modification effect was checked using 10× higher pCMBS or 100 s pCMBS exposure. Modification rate analysis was performed on data from individual oocytes. For β2N265C receptor modification, spontaneous current after modification was plotted against [pCMBS] × time and fitted with single exponential functions. For α1M236C modification, the ratio of low GABA to high GABA responses were calculated, normalized to the pre-modification control, and plotted against [pCMBS] × time (mM×s). Linear least squares fits to the first three to five points were used to determine the initial modification rate in M^−1^s^−1^. A subset of oocytes were modified first in the absence of etomidate, then in the presence of etomidate. Modification rates in both conditions were independently fitted with linear least squares.

### Electrophysiological Data Analysis

Analyses for agonist concentration-responses, etomidate-induced left shift, and allosteric co-agonist model fitting followed our approach described elsewhere [16], [27]. Experimental peak currents were normalized to maximal GABA responses, and GABA concentration-response data for individual oocytes in the absence and presence of etomidate were fitted with logistic functions using non-linear least squares (Prism v.5, Graphpad Software):
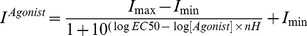
(1)where EC_50_ is the half-maximal activating concentration and nH is Hill slope. Etomidate-dependent direct activation of receptors was analyzed similarly.

EC_50_ shift ratios were calculated from the difference in log(GABA EC_50_) values (Δlog(EC_50_)) measured in the presence of 3.2 µM etomidate versus control.

PTX-sensitive leak currents (I_PTX_) normalized to 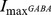 (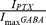) provided estimates of basal open probability. GABA efficacy was estimated based on enhancement of maximal GABA responses by etomidate or alphaxalone [27].

The estimated fraction of activated receptors, 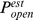 corrected for both basal activity and maximal GABA efficacy, was calculated as previously described [35]:
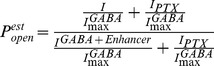
(2)


A Monod-Wyman-Changeux (MWC) co-agonist mechanism with two equivalent sites each for GABA and etomidate (Fig. 1D; Eq. 3) was fitted by non-linear least squares to 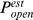 values derived from GABA concentration-responses with and without etomidate as well as etomidate direct activation. Both [GABA] and [ETO] were independent variables:
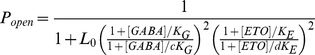
(3)


In Eq. 3, L_0_ is a dimensionless basal closed:open gating equilibrium variable, K_G_ and K_E_ are dissociation constants for respectively, GABA and etomidate binding to inactive receptors, and c and d are the respective (dimensionless) ratios of dissociation constants in activated versus inactive receptors. The maximal agonist efficacies of GABA and etomidate are inversely related to, respectively, L_0_c^2^ and L_0_d^2^.

### Estimation of etomidate binding affinity from cysteine protection

Because α1M236C is fully protectable by etomidate [12] or propofol, anesthetic-dependent inhibition of the α1M236C modification rate reflects anesthetic site occupancy, as we reported for β2M286C [14]. We therefore plotted α1M236C modification rate results as a function of etomidate concentration and fitted logistic functions (Eq. 1) to the data using non-linear least squares with a Hill slope of 1.0. The 50% protection concentration (PC_50_) is assumed to be the anesthetic dissociation constant (K_E_).

### Structural Homology Modeling

A model of the human α1β3γ2 GABA_A_ receptor was constructed using the Prime module in Schrödinger Maestro 9.7 (release 2014-1, run on the SBGrid Consortium cluster housed at Harvard Medical School, Boston, MA). The structure was based on the GluCl structure template PDB 3RHW [20] after removal of co-crystallized ivermectin and antibody fragments. Separate amino acid sequence alignments of each GABA_A_ subunit to GluCl were performed using the ClustalW algorithm. Alignments were gap-free in all M1 through M3 domains. The intracellular ends of GABA_A_ subunit M3 and M4 were established by the strong alignment of homologous sequences in the middle of these domains and the length of the helices in the GluCl crystal structure. Intervening M3–M4 loops of GABA_A_ subunit sequences were then truncated and replaced with that of the modified GluCl used for crystallography. Alignment introduced a modest number of gaps into extracellular domains and M4 sequences. Some of these were edited out or moved to preserve local GluCl secondary structure. The final sequence alignments (Figure S1) are similar to those of Bertaccini et al [28]. Subunit monomer models were built using the “knowledge-based methods” option in Prime, and the heteropentamer model was assembled from five subunit monomer models in the established pentameric arrangement [6]. Model refinement used VSGB solvation [36] and a simulated membrane forcefield (dielectric = 80) bracketing the transmembrane helices. Stepwise structural energy minimization proceeded first with non-template loops, then non-conserved sidechain rotamers, and finally full all-atom minimization. Molecular graphics images were produced using UCSF Chimera software version 1.8.1.

### Statistical Analysis

Results are reported as mean ± standard deviation unless otherwise noted. Statistical comparisons of results for three or more groups were performed using ANOVA with Tukey’s *post-hoc* test. Pairwise comparisons were performed using Students t-tests. Statistical significance was inferred at p<0.05.

## Results

### Structural Homology Modeling

A homology model for α1β3γ2L GABA_A_ receptors based on GluCl is depicted in Fig. 1, panels A through C. This model is similar to other GABA_A_ receptor homology models based on both GLIC and GluCl [9], [12], [28], [37]. The transmembrane structure of β3 subunits in our model closely matches that of crystallized β3 homomeric GABA_A_ (PDB 4COF) [7]. Residues thought to contribute to etomidate or propofol binding (αL232, αM236, αT237, αI239, βM286, and βV290) all appear in or near the β+/α− transmembrane interfacial pockets of the model structure. In addition, βN265 (M2-15′) is also adjacent to the β+/α− interface of the model.

### Functional characteristics of α1β2N265Cγ2L GABA_A_ receptors

**Figure 2:**
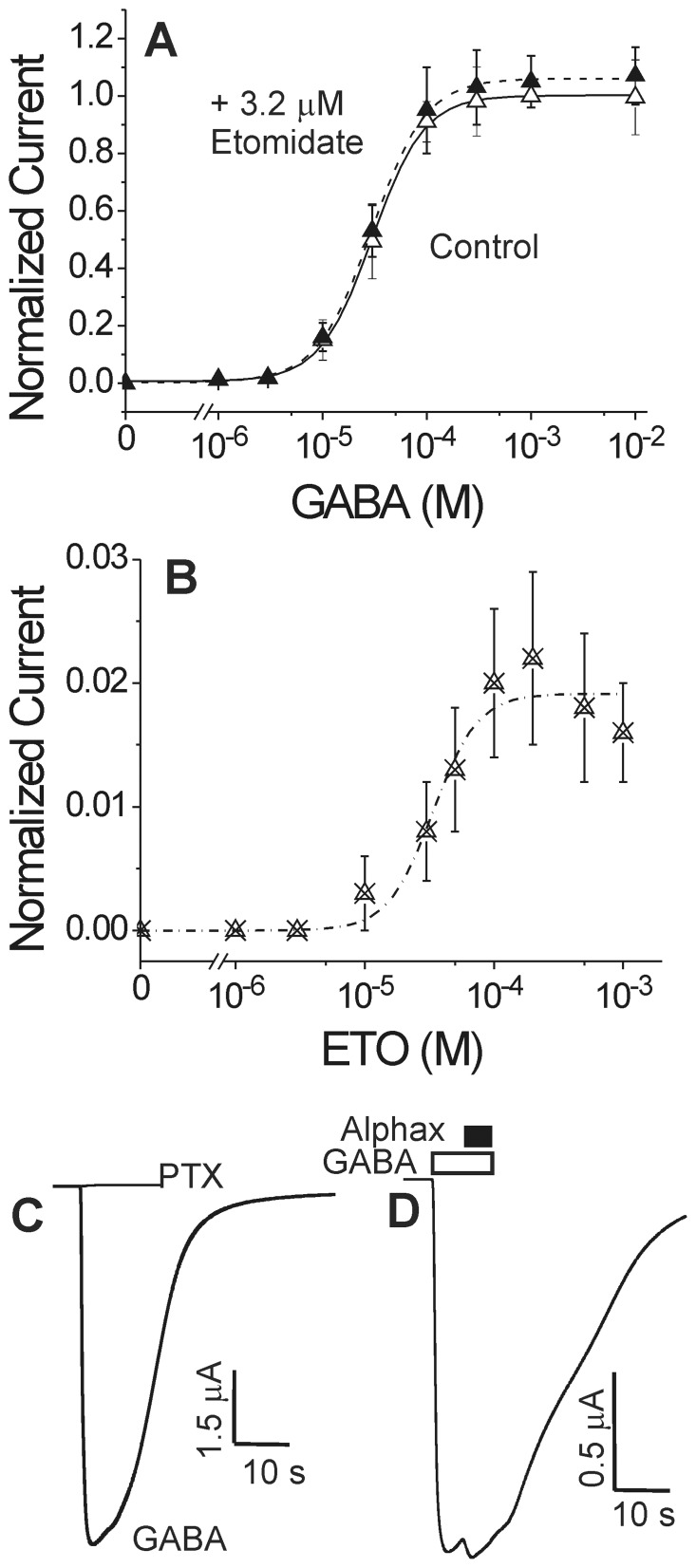
Electrophysiological characterization of α1β2N265Cγ2L GABA_A_ receptors. **Panel A:** GABA concentration response in oocytes. Data points are mean ± sd (n ≥4) peak currents normalized to maximal GABA (1 mM) responses. Lines through data represent fits to logistic equations (Eq. 1, Methods). Open symbols: GABA alone; EC_50_ = 31±4.7 µM; nH = 1.75±0.088. Filled symbols: GABA plus 3.2 µM etomidate; EC_50_ = 30±3.2; nH = 1.67±0.068; maximum response = 1.06±0.01. **Panel B:** Etomidate agonism concentration response in oocytes. Data points are mean ± sd (n ≥4) peak currents normalized to maximal GABA (1 mM) responses. The line represents a logistic fit. EC_50_ = 35±5.7 µM; nH = 2.5±1.1; maximum response = 0.020±0.007. **Panel C:** Spontaneous channel gating activity in an oocyte, assessed using picrotoxin (PTX). No outward current during PTX application was observed. Current elicited with 1 mM GABA in the same cell is also displayed. **Panel D:** Estimation of maximal GABA efficacy in oocytes. GABA (3 mM; white bar) alone elicits a current that is enhanced about 5% with co-application of alphaxalone (2 µM; black bar). Average results for GABA efficacy are reported in Table 1.

Our initial approach to testing steric interactions between βN265 and etomidate followed the substituted cysteine modification and protection strategy that we and others have used to test anesthetic interactions with GABA_A_ receptor transmembrane domains [12]–[14], [32]. We electrophysiologically characterized α1β2N265Cγ2L GABA_A_ receptors for spontaneous activity, GABA EC_50_ and efficacy, and sensitivity to both etomidate modulation and direct activation (Fig. 2). Oocytes expressing α1β2N265Cγ2L receptors produced GABA-activated chloride currents with GABA EC_50_ similar to α1β2γ2L receptors (Fig. 2A, solid symbols, Table 1). However, etomidate at a concentration that produces general anesthesia in tadpoles (3.2 µM) did not significantly enhance GABA-activated α1β2N265Cγ2L currents and did not alter GABA EC_50_ (Fig. 2A, open symbols). High concentrations of etomidate (>30 µM) elicited very small currents with maximal amplitudes around 1 to 2% of maximal GABA responses (Fig. 2B), consistent with a prior report by McCracken et al [32]. Picrotoxin-sensitive spontaneous chloride leak in α1β2N265Cγ2L receptors was not detectable (Fig. 2C) and, thus less than experimental noise (∼0.2% of maximal GABA-elicited currents), indicating a very low spontaneous opening probability. In contrast to etomidate, alphaxalone (2 µM) strongly modulated low GABA responses in α1β2N265Cγ2L receptors, and enhanced maximal GABA currents by 5 to 10% (Fig. 2D). This indicates that over 90% of α1β2N265Cγ2L receptors are activated at GABA concentrations of 1 to 10 mM, which is slightly higher than GABA efficacy estimates for α1β2γ2L (Table 1). Thus, α1β2N265Cγ2L receptors, like α1β2N265Mγ2L [27], display a functional phenotype with basal activity and GABA sensitivity similar to wild-type receptors, but extremely low sensitivity to etomidate. These functional data do not indicate whether insensitivity to etomidate is due to low drug affinity, low drug efficacy, or both.

**Table 1:** Wild-type and Mutant GABA_A_ receptor functional characteristics in *Xenopus* oocytes.

Receptor	GABA EC_50_ (µM)	Max. GABA Efficacy a	ETO EC_50_ (µM)	Max. ETO Efficacy a	Spontaneous Activity b	EC_50_ Ratio (Ctl/3.2 ETO) c
α1β2γ2L	34±7	0.85±0.04	30±9.2	0.35±0.06	<0.002	17±3.5
	(n = 4)	(n = 4)	(n = 4)	(n = 6)	(n = 4)	(n = 4)
α1β2N265Cγ2L	31±5	0.91±0.05	35+5.7	0.02±0.006 **	<0.002 **	1.0±0.16 **
	(n = 4)	(n = 4)	(n = 3)	(n = 3)	(n = 5)	(n = 4)
β3-α1M236C/β3-α1M236C-γ2L	58±4.6*	0.63±0.08	40+3.9	0.60±0.10	<0.002	18±2.3
	(n = 4)	(n = 4)	(n = 3)	(n = 3)	(n = 3)	(n = 3)
β3N265M-α1M236C/β3N265M-α1M236C-γ2L	110±15**	0.60±0.12	n.a.	<0.002 **	<0.002	1.0±0.16 **
	(n = 3)	(n = 3)	(n = 3)	(n = 3)	(n = 3)	(n = 3)

a Efficacy is the estimated fraction of activatable receptors that open in the presence of an agonist (either GABA or etomidate). The detection limit for etomidate agonist efficacy is about 0.2% of maximal GABA response.

b Spontaneous activity is the estimated fraction of active receptors in the absence of either GABA or etomidate. The detection limit for spontaneous activity is about 0.2% of maximal GABA response.

c EC_50_ ratio is the ratio of control GABA EC_50_ to GABA EC_50_ measured in the presence of 3.2 µM etomidate. Large ratios indicate sensitivity to etomidate modulation.

*Differs from wild-type value at p<0.05. **Differs from wild-type value at p<0.01.

### Etomidate does not protect β2N265C from pCMBS modification

**Figure 3:**
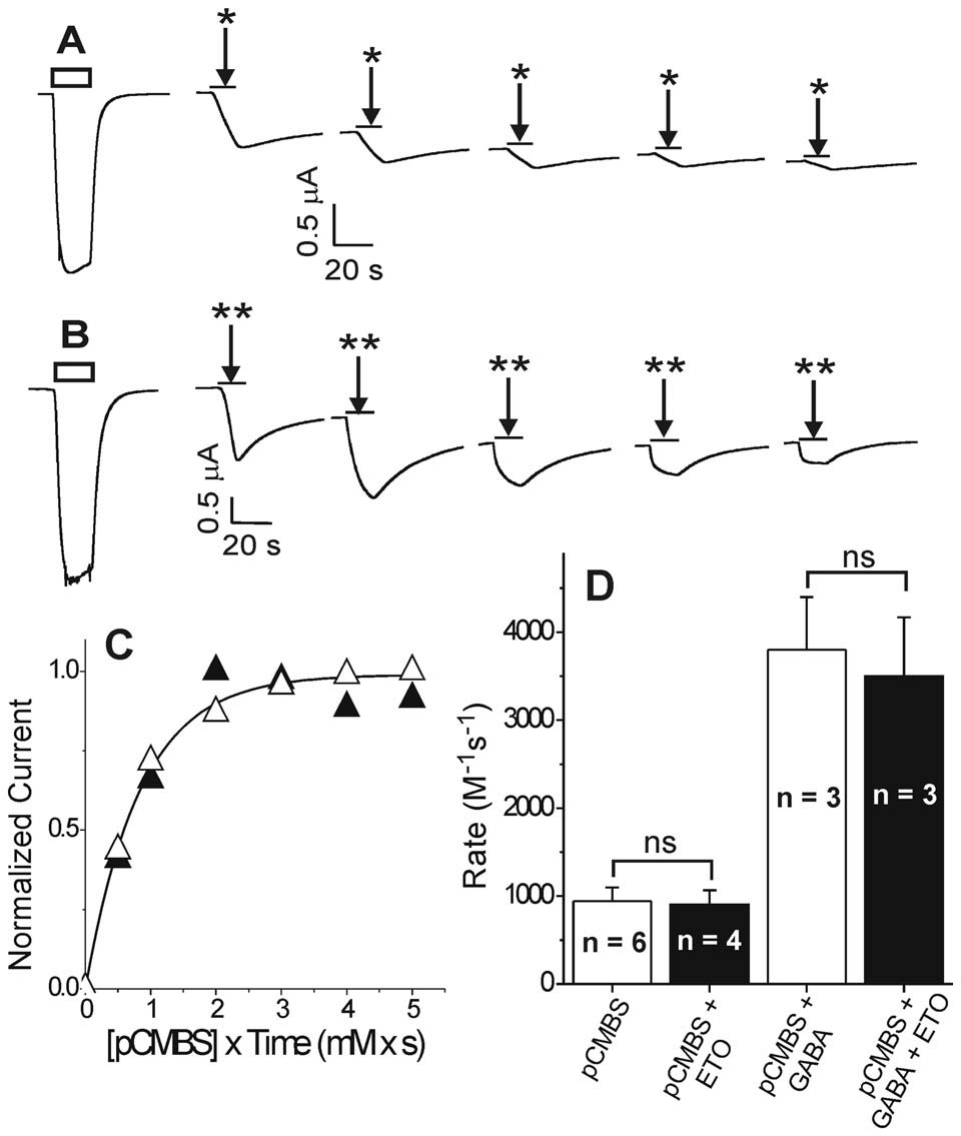
pCMBS modification and lack of etomidate protection in oocyte-expressed α1β2N265Cγ2L GABA_A_ receptors. **Panel A:** The first trace is elicited with EC_10_ GABA (10 µM; white bar) and subsequent traces were recorded during sequential 10 s exposures to 0.1 mM *p*-chloromercuribenzenesulfonate (pCMBS; arrows; *), followed by wash. Basal currents increase with incremental exposure to pCMBS. **Panel B:** Traces are from another oocyte. Arrows (**) indicate 10 s exposures to 0.1 mM pCMBS plus 300 µM etomidate, followed by wash. **Panel C:** Rate analysis of current data from panels A (open symbols) and B (solid symbols) is shown, plotted against cumulative pCMBS exposure time. The line represents a nonlinear least squares single exponential fit to control data (no etomidate). The fitted rate constant is 1200±50 M^−1^s^−1^. The fitted rate for protection data (+ etomidate) is 1300±270 M^−1^s^−1^. **Panel D:** Average ± sd pCMBS modification rates in the absence and presence of 1 mM GABA and/or 300 µM etomidate.

Exposing α1β2N265Cγ2L GABA_A_ receptors to a water soluble thiol-reactive probe, para-chloromercuribenzene sulfonate (pCMBS), produced currents that did not fully reverse with pCMBS washout (Fig. 3A). These currents were blocked by picrotoxin (not shown), indicating that covalent modification of β2N265C with pCMBS irreversibly activated α1β2N265Cγ2L channels. Plotting the post-wash current against cumulative pCMBS exposure resulted in an average apparent modification rate of 950±155 M^−1^s^−1^ (n = 6). Addition of GABA increased the apparent rate of modification to 3800±600 M^−1^s^−1^, (n = 3). In contrast, control experiments in wild-type α1β2γ2L receptors revealed no functional effects of 2 mM pCMBS exposures up to 60 s (120 mM×s; n = 5; data not shown).

Absent any data to guide estimation of etomidate affinity in α1β2N265Cγ2L receptors, we tested protection using a range of concentrations up to 300 µM, which fully protected β2M286C from modification in both the absence and presence of GABA [14]. However, in α1β2N265Cγ2L receptors, co-administration of pCMBS with etomidate at up to 300 µM, with or without GABA, did not alter the rate or extent of the pCMBS-receptor reaction (Fig. 3, B–D).

There are several hypotheses for this lack of etomidate protection at βN265C: 1) etomidate may bind normally, but not near the βN265 residue, implying that the βN265C mutation eliminates drug transduction/efficacy; 2) the βN265C mutation may reduce affinity for etomidate so that site occupancy remains low at concentrations up to 300 µM; and 3) etomidate may bind near βN265, but without obstructing pCMBS access. To address some of these possibilities, we assessed the effects of combining βN265M with previously characterized second mutations in the etomidate site. In order to reduce potential receptor assembly variations caused by subunit-subunit interfacial mutations, we also used concatenated dimer and trimer subunit assemblies for these double-mutant experiments.

### A β+/α− site occupancy reporter reveals that βN265M mutations reduce etomidate affinity

**Figure 4:**
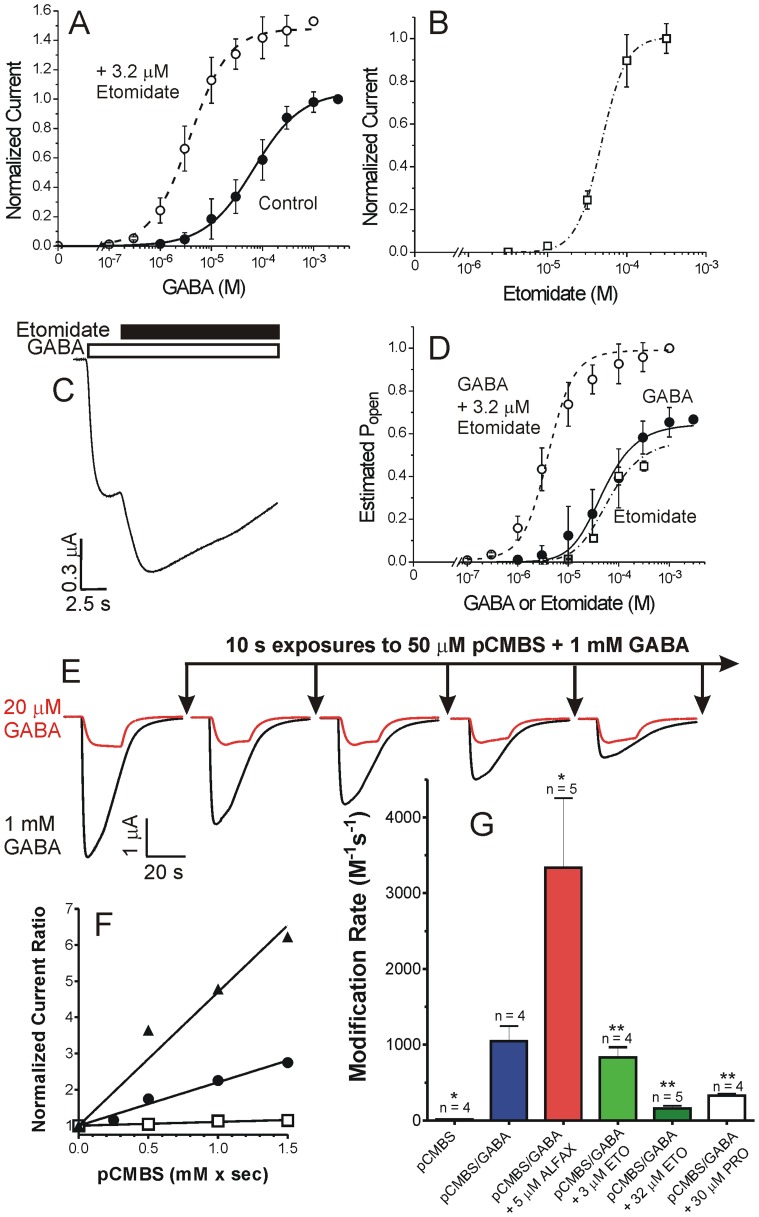
Modification and protection at α1M236C reflects anesthetic site occupancy. **Panel A)** Data represent mean ± SD peak current responses to GABA from oocytes (n = 4) expressing β3-α1M236C/β3-α1M236C-γ2L GABA_A_ receptors, normalized to maximal GABA responses. Lines represent logistic fits to responses using GABA alone (solid circles; EC_50_ = 58 µM) and GABA with 3.2 µM etomidate (open circles; EC_50_ = 3.2 µM). **Panel B)** Data represent mean ± SD peak current responses to etomidate from oocytes (n = 3) expressing β3-α1M236C/β3-α1M236C-γ2L GABA_A_ receptors, normalized to maximal GABA responses. The line represents a logistic fit with etomidate EC_50_ = 47 µM. **Panel C)** A single voltage-clamp current trace illustrating maximal GABA (3 mM; white bar above trace) efficacy in oocyte-expressed β3-α1M236C/β3-α1M236C-γ2L GABA_A_ receptors, enhanced with addition of 10 µM etomidate (black bar). **Panel D)** Data from panels A and B were renormalized to maximal GABA efficacy (methods; Eq. 2) and fitted with a global MWC equilibrium co-agonist equation (methods; Eq. 3). Lines through data points represents the fitted MWC model: L_0_ = 10,000; K_G_ = 42±8.7 µM; c = 0.0075±0.00048; K_E_ = 50±12 µM; d = 0.0089±0.00096. **Panel E)** Traces are from a single oocyte expressing β3-α1M236C/β3-α1M236C-γ2L GABA_A_ receptors, demonstrating the effects of repeated pCMBS applications on the relative responses to low versus high GABA stimulation. **Panel F)** Points represent response ratios to low (EC10) vs. high GABA, normalized to pre-modification control values. Lines through data represent linear fits used to determine relative bimolecular modification rates: GABA+pCMBS (circles; 1200±57 M^−1^s^−1^); GABA+pCMBS +5 µM alphaxalone (triangles; 3700±270 M^−1^s^−1^); and GABA+pCMBS +32 µM etomidate (squares; 106±8.4 M^−1^s^−1^). **Panel G)** Summary of modification rate results (mean ± se) for all oocytes expressing β3-α1M236C/β3-α1M236C-γ2L GABA_A_ receptors under different conditions. The rate with pCMBS alone is significantly accelerated with addition of GABA and GABA/alphaxalone. Relative to GABA+alphaxalone, modification in the presence of GABA is slowed 65% by 3 µM etomidate, 95% by 32 µM etomidate, and 91% by 30 µM propofol. * p<0.05; ** p<0.01.

**Figure 5:**
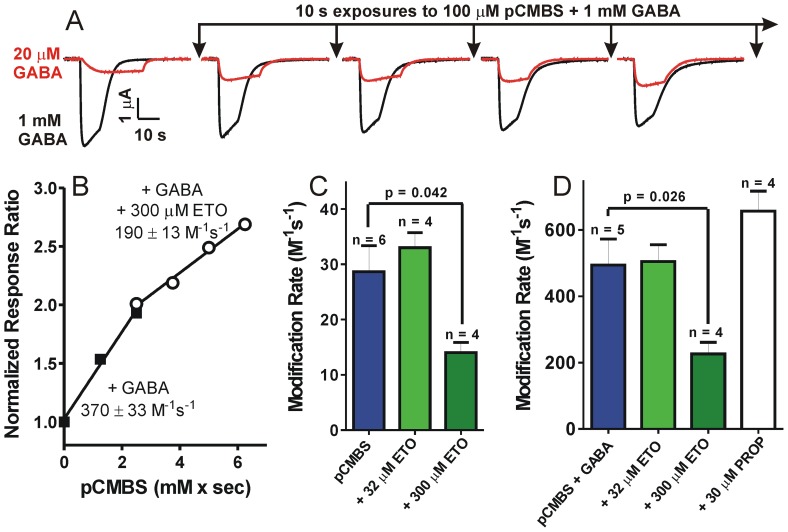
The βN265M mutation reduces anesthetic-dependent site occupancy. **Panel A)** Voltage-clamp current traces are from a single oocyte expressing β3N265M-α1M236C/β3N265M-α1M236C-γ2L GABA_A_ receptors, showing the effects of repeated pCMBS+GABA applications on responses to low versus high GABA. **Panel** **B)** Points represent response ratios to low (EC10) vs. high GABA, normalized to pre-modification control values, for one oocytes exposed to: GABA+pCMBS (solid squares) followed by GABA+pCMBS +300 µM etomidate (open circles). Lines through data represent linear fits; labels are the apparent bimolecular modification rates (slopes). **Panel** **C)** Summary of modification rate results for all oocytes expressing β3N265M-α1M236C/β3N265M-α1M236C-γ2L GABA_A_ receptors modified with pCMBS, in the absence vs. presence of etomidate. Etomidate (300 µM) reduced the apparent modification rate by 51%. **Panel** **D)** Summary of modification rate results for GABA-activated receptors (modified in the presence of 1 mM GABA). Modification is inhibited 54% by 300 µM etomidate. Propofol (30 µM) does not significantly inhibit modification.

We previously reported that GABA_A_ receptors with cysteine substitutions at α1M236 are modified by pCMBS and that bound etomidate blocks α1M236C modification [12]. Thus, as we showed for βM286C [14], anesthetic-dependent reduction in the α1M236C modification rate reflects β+/α− site occupation. For the experiments described here, we preferred α1M236C as a site occupancy reporter because, unlike βM286C, it maintains etomidate sensitivity [12]. We first demonstrated that, in GABA_A_ receptors assembled from concatenated subunit dimers and trimers, α1M236C is both pCMBS modifiable and protected by both etomidate and propofol (Fig. 4). We then examined anesthetic protection at α1M236C in receptors assembled from concatenated dimers and trimers with βN265M mutations, for comparison (Fig. 5).

Voltage-clamped oocytes expressing β3-α1M236C dimers and β3-α1M236C-γ2L trimers produced GABA-activatable receptor-channels that were both modulated and directly activated by etomidate (Fig. 4A, B; Table 1). Etomidate efficacy as an agonist was similar to that of GABA (Fig. 4B, which was 63±4.4% (n = 3) based on enhancement of maximal currents at high GABA (Fig. 4A, C). These pharmacological responses were quantitatively consistent with an MWC allosteric co-agonist model (Fig. 4D). Exposing β3-α1M236C/β3-α1M236C-γ2L receptors to pCMBS irreversibly increased their sensitivity to low GABA (20 µM ≈ EC10–20) relative to high GABA, indicating enhanced channel gating (Fig. 4E). The apparent rate of α1M236C modification increased about 3-fold when pCMBS was co-applied with GABA, and was further accelerated by addition of alphaxalone, a positive allosteric modulator that does not interact with etomidate or propofol sites (Fig. 4F, G). Because etomidate directly activates β3-α1M236C/β3-α1M236C-γ2L receptors, we did not test etomidate protection in the absence of GABA. Etomidate at 3 µM did not significantly alter the apparent rate of α1M236C modification in GABA-activated receptors (Fig. 4G). We hypothesized that this result reflected two opposing effects of etomidate, as we have previously reported [12], [14]: etomidate both increased the fraction of activated channels relative to GABA alone (see Figs. 4A, C), while also blocking pCMBS access. Therefore, we used the GABA+alphaxalone modification rate as a control for analysis of etomidate-dependent protection results, because the fraction of activated receptors was maximized when GABA was co-applied with either alphaxalone or etomidate. Relative to the rate observed with GABA plus alphaxalone, 3.2 µM etomidate slowed modification about three-fold, and 32 µM etomidate reduced the modification rate over twenty-fold (Fig. 4G). Logistic analysis of these modification rate data indicated 50% protection at 1.07 µM etomidate (PC_50_; 95% CI = 0.24 to 4.7 µM).

Double-mutant β3N265M-α1M236C/β3N265M-α1M236C-γ2L GABA_A_ receptors produced GABA-activated currents that were insensitive to both etomidate modulation and direct etomidate activation (Table 1). Currents mediated by β3N265M-α1M236C/β3N265M-α1M236C-γ2L double-mutant receptors also demonstrated cumulative irreversible gating changes following repeated exposures to pCMBS (Fig. 5A). Modification by pCMBS was accelerated over 10-fold in the presence of GABA. Because β3N265M-α1M236C/β3N265M-α1M236C-γ2L receptors were etomidate insensitive, we were able to study etomidate protection at α1M236C in both the absence and presence of GABA (i.e. inactive vs. mostly activated channels). Insensitivity to etomidate also enabled sequential measurement in the same oocytes of modification rates in the absence vs. presence of etomidate, (e.g. Fig. 5B). Etomidate at 32 µM did not significantly alter the apparent rate of α1M236C modification in either the absence (Fig. 5C) or presence of GABA (Fig. 5D). Using 300 µM etomidate, rates of modification were reduced by about two-fold from control in both conditions (Fig. 5B–D). Logistic fits to these data estimated that in the absence of GABA etomidate PC_50_ ≈ 300 µM (95% CI = 215 to 423 µM) and is similar in the presence of GABA (PC_50_ ≈ 290 µM; 95% CI = 206 to 412 µM). Assuming that the PC_50_ estimates reflect etomidate occupancy, we infer that etomidate binds very weakly (dissociation constant ≈ 300 µM) to both inactive and GABA-activated β3N265M-α1M236C/β3N265M-α1M236C-γ2L receptors, in comparison to activated β3-α1M236C/β3-α1M236C-γ2L receptors (dissociation constant ≈ 1 µM).

### Propofol occupancy of β+/α− sites is also reduced by βN265M mutations

We also demonstrated that propofol at 30 µM, like etomidate, reduced the rate of pCMBS modification at α1M236C by about 90% in activated free subunit α1M236Cβ3γ2L receptors (not shown) and in concatenated β3-α1M236C/β3-α1M236C-γ2L receptors (Fig. 4G). In comparison, 30 µM propofol did not reduce the rate of pCMBS modification in GABA-activated β3N265M-α1M236C/β3N265M-α1M236C-γ2L receptors (Fig. 5D), indicating very low β+/α− site occupancy.

### A β+/α− site mutation that mimics bound anesthetic reveals that βN265M impairs transduction

To study the effect of βN265M mutations on β+/α− site transduction, without the need to establish drug site occupancy, we used another mutation. The azi-etomidate photolabeled α1M236 sidechain abuts the β+/α− cleft where etomidate binds, and α1M236W mutation mimics the positive allosteric effects of anesthetic binding while reducing etomidate modulation [16]. Thus, the tryptophan substituted sidechain at α1M236 likely occupies the same space as bound etomidate, and shares transduction mechanisms that enhance channel gating.

**Figure 6:**
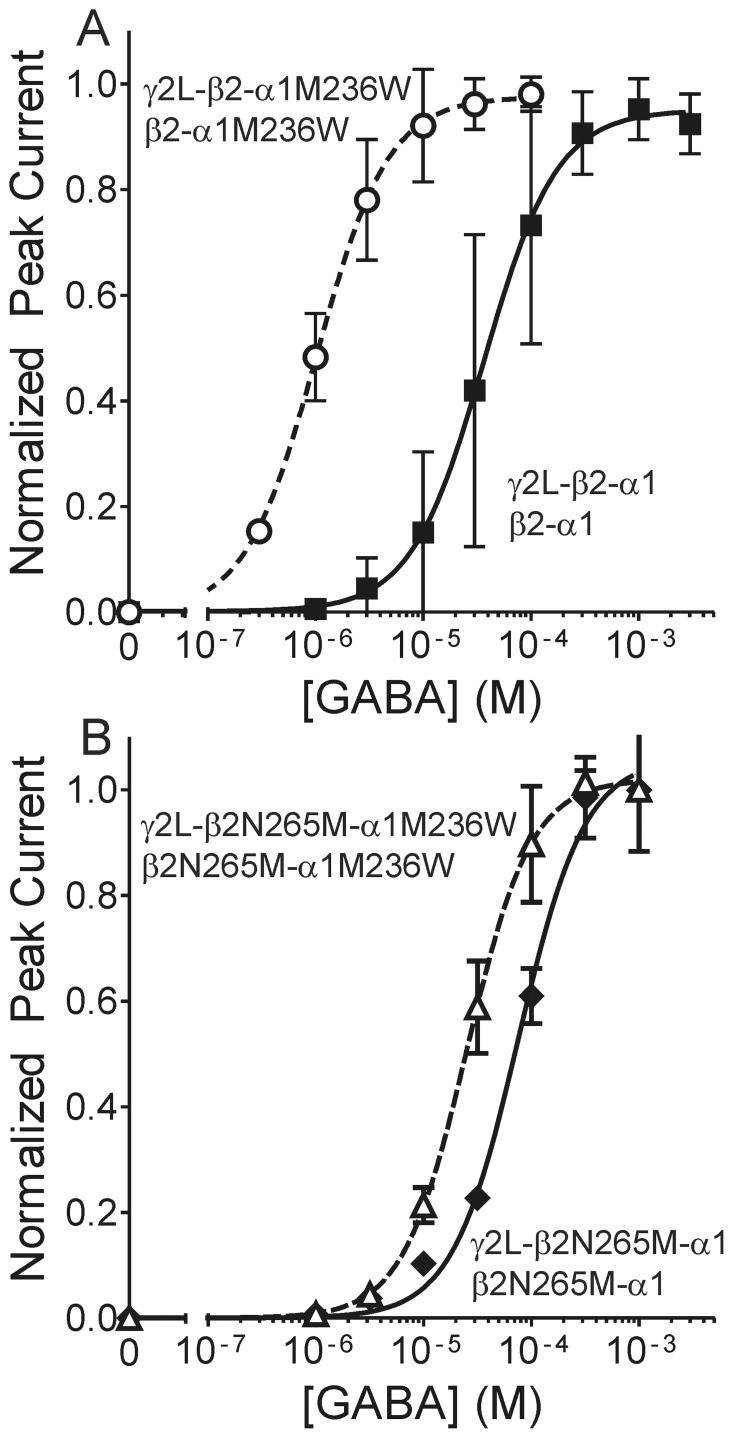
The βN265M mutation reduces the channel gating effects of a α1M236W mutation that mimics etomidate. **Panel A)** Data are reproduced from Guitchounts et al [38] showing mean ± SD GABA concentration-response, normalized to maximal currents, from oocytes expressing concatenated receptor dimer and trimer constructs. Lines through data points represent non-linear least squares logistic fits (Eq. 1, methods). Solid squares represent β2-α1/γ2L-β2-α1 receptors (GABA EC_50_ = 36 µM) and open circles represent β2-α1M236W/γ2L-β2-α1M236W receptors (EC_50_ = 1.0 µM). **Panel** **B)** Data points are mean ± SD current responses to GABA, normalized to maximal currents. Solid diamonds represent β2N265M-α1/γ2L-β2N265M-α1 receptors (GABA EC_50_ = 76 µM; 95% CI = 63 to 92 µM) and open triangles represent β2N265M-α1M236W/γ2L-β2N265M-α1M236W- receptors (EC_50_ = 25 µM; 95% CI = 22 to 29 µM).

In α1β2γ2L receptors formed from free subunits, α1M236W produced a 20-fold reduction in GABA EC_50_ [16]. In receptors where pentameric subunit assembly is constrained by co-expression of concatenated β2-α1 dimers and γ2L-β2-α1 trimers, α1M236W mutations in both subunit assemblies produced a 36-fold reduction in GABA EC_50_ (Fig. 6A) [38]. We introduced β2N265M mutations into both dimer and trimer concatenated subunit assemblies, and then added α1M236W mutations to both constructs. GABA concentration response data from β2N265M-α1/γ2L-β2N265M-α1 receptors resulted in GABA EC_50_ = 76 µM; n = 4 oocytes; Fig. 6B). This represents about a two-fold increase in GABA EC_50_ relative to β2-α1/γ2L-β2-α1 receptors, consistent with previous studies of βN265M [25], [27], [39]. Etomidate neither modulated nor directly activated β2N265M-α1/γ2L-β2N265M-α1 receptors (4 cells tested for each effect, not shown). GABA concentration-responses for β2N265M-α1M236W/γ2L-β2N265M-α1M236W (n = 4 oocytes) were characterized by GABA EC_50_ = 25 µM, three-fold lower than the β2N265M-α1/γ2L-β2N265M-α1 value (Fig. 6B). Thus, α1M236W produced a small but significant channel gating effect when βN265M mutations were present. The GABA EC_50_ shift produced by α1M236W when βN265M was present was about 10-fold smaller than the shift in the wild-type receptor background. In other words, βN265M mutations dramatically reduced, but did not eliminate β+/α− site transduction.

## Discussion

### Major findings

We used both direct and indirect structure-function strategies to investigate the role of the GABA_A_ receptor βN265 (M2-15′) residue in the binding and efficacy of etomidate and propofol within their established β+/α− interfacial sites. Cysteine substitution at βN265 produced receptors that were profoundly insensitive to etomidate (Fig. 2). Moreover, etomidate at concentrations up to 300 µM did not protect βN265C from covalent thiol modification (Fig. 3). The absence of etomidate effects obscures whether βN265C alters anesthetic binding vs. efficacy. However, the impact of βN265M mutations was revealed in studies of receptors containing second “reporter” mutations within the β+/α− anesthetic sites. These indirect experiments show that βN265M mutations both weaken anesthetic binding and impair β+/α− site transduction (efficacy).

### Anesthetic binding versus efficacy in GABA_A_ receptors

The challenge of distinguishing whether ligand-gated ion channel mutations affect agonist binding, efficacy, or both, is well-known [40]. Both etomidate and propofol are allosteric agonists at GABA_A_ receptors [1], [2]. Within the MWC mechanistic framework, mutations can affect apparent sensitivity to anesthetics in three ways [41]: 1) altering the basal inactive-active equilibrium (L_0_ in Eq. 3), 2) altering anesthetic binding to inactive receptors (K_E_), and 3) altering anesthetic efficacy (d), which is equivalent to selectively altering binding to activated receptor states (dK_E_).

### The βN265M mutation reduces both etomidate efficacy and binding affinity

**Figure 7:**
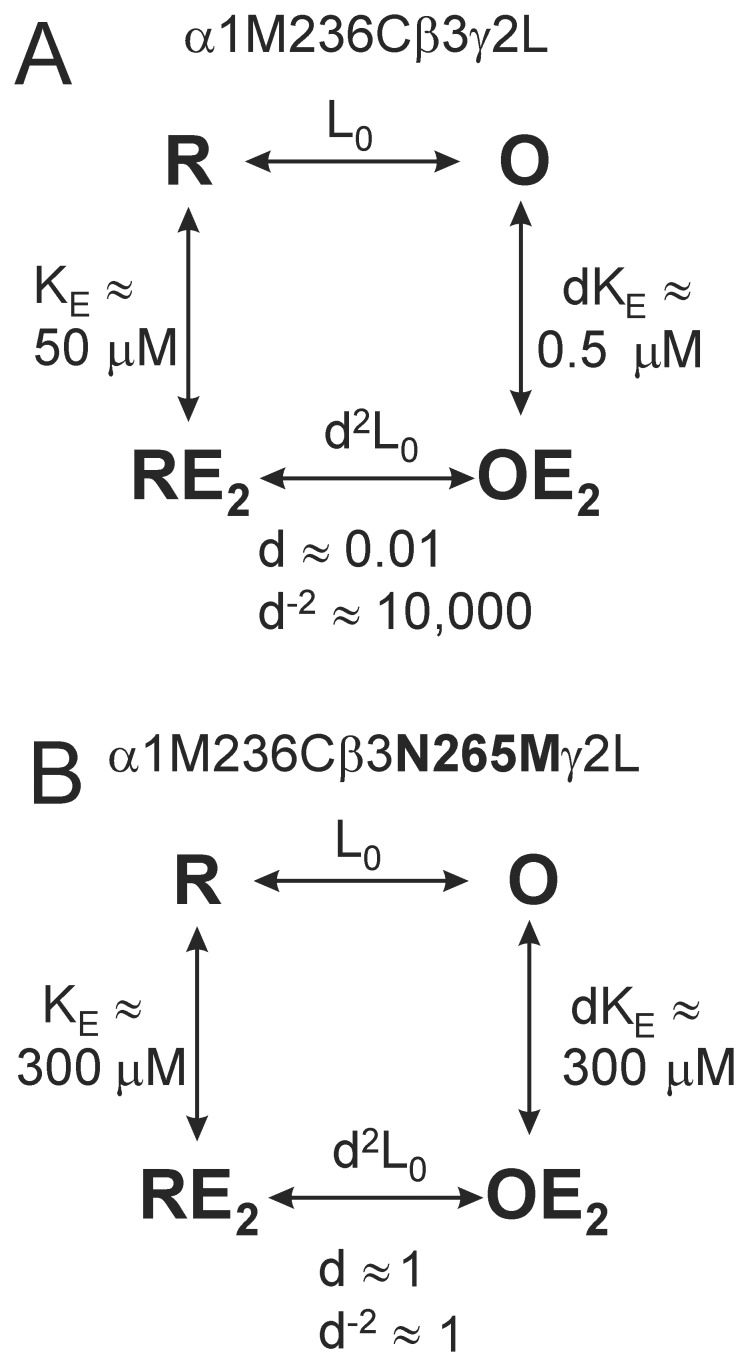
Effects of βN265M mutation on etomidate in inactive versus active GABA_A_ receptors. Monod-Wyman-Changeux equilibrium allosteric models are diagrammed for both α1M236Cβ2γ2L (A) and α1M236Cβ2N265Mγ2L (B) receptors, each with two equivalent etomidate sites. Etomidate dissociation constants for both inactive (R; K_E_) and GABA-activated (O; dK_E_) receptors are rounded estimates based on both functional analysis and modification/protection results (Figs. 6 and 7). Etomidate-sensitive α1M236Cβ2γ2L receptors bind etomidate 100-fold more avidly in the active vs. inactive state, resulting in a 10,000-fold shift in the open-closed equilibrium constant (d^2^L_0_ vs. L_0_) when both sites are occupied. In contrast, etomidate-insensitive α1M236Cβ2N265Mγ2L receptors display low affinity (K_E_ ≈ dK_E_ ≈ 300 µM) for etomidate in both resting and GABA-activated states. Thus, high etomidate concentrations result in only partial site occupancy and weak modulatory effects.

Both current and previous [25], [27], [32] functional studies reveal that βN265C and βN265M mutations eliminate etomidate sensitivity, providing no information regarding changes in drug binding or efficacy. To investigate drug occupancy (binding) of β+/α− anesthetic sites independent of drug effects, we used a modifiable and protectable reporter cysteine, α1M236C. Concatenated β3-α1M236C/β3-α1M236C-γ2L receptors, like free subunit α1M236Cβ2γ2L receptors [12], retained sensitivity to both etomidate modulation and direct activation. MWC model analysis indicated that the respective dissociation constants for etomidate in inactive (K_E_) and active (dK_E_) receptors are approximately 50 µM and 0.5 µM (Figs. 4D, 7A). Thus, etomidate allosteric efficacy (d) in this receptor is approximately 0.01 (Figs. 4D, 7A), comparable to estimates for both wild-type receptors [1] and free subunit α1M236Cβ2γ2L receptors [12]. Etomidate occupation of its two sites per β3-α1M236C/β3-α1M236C-γ2L receptor is thus predicted to shift the closed:open equilibrium by d^−2^ ≈ 10,000-fold toward open states (Fig. 7A). This accounts for the robust etomidate agonism observed in these channels (Fig. 4B). The MWC estimate for etomidate affinity in activated β3-α1M236C/β3-α1M236C-γ2L receptors (dK_E_ ≈ 0.5 µM) is also in good agreement with that for free-subunit α1M236Cβ2γ2L receptors(dK_E_ ≈ 1 µM) [12] and our current etomidate protection results (PC_50_ ≈ 1 µM; Fig. 5).

In comparison, protection experiments in double-mutant concatenated β3N265M-α1M236C/β3N265M-α1M236C-γ2L receptors indicated only ∼50% occupancy at 300 µM etomidate, implying extremely low-affinity binding to both inactive (K_E_ ≈ 300 µM) and GABA-activated (dK_E_ ≈ 300 µM) receptors (Fig. 7B). Etomidate’s similar affinity for both inactive and active receptors in the double mutant receptors implies an allosteric efficacy near 1.0 (Fig. 7B). This means that etomidate binding to receptors with βN265M mutations doesn’t shift the closed:open state distribution, consistent with the observed absence of drug effects on channel activity. This estimated effect of βN265M on etomidate efficacy (10,000-fold reduction for two sites) is much larger than the efficacy effect (25-fold reduction for two sites) that was previously estimated for β2N265S mutations [27]. Comparing results for α1M236Cβ3γ2L and α1M236Cβ3N265Mγ2L receptors (Fig. 7) suggests that methionine substitution reduces etomidate affinity for inactive receptors about 6-fold (K_E_ ≈ 50 µM vs. 300 µM), while efficacy (d) is weakened about 100-fold per site. Together, reductions of both drug affinity and efficacy account for the approximately 600-fold reduction in apparent etomidate binding affinity for GABA-activated receptors (dK_E_ ≈ 0.5 µM vs. 300 µM).

Our evidence demonstrating reduced etomidate affinity in both inactive and GABA-activated receptors is consistent with a direct role for βN265 in drug binding. This interaction is also suggested by *in silico* docking calculations [12]. Additional indirect support for this hypothesis comes from studies of M2-15′ residues on other pLGICs that contact alcohols and anesthetics [30], [39], [42]. In addition, other outer M2 domain residues may also contribute to anesthetic sites in GABA_A_ receptors [31] and related pLGICs [30], [43], [44]. However, the indirect nature of our evidence cannot rule out the possibility that βN265 mutations indirectly influence etomidate binding.

### The propofol sites in β+/α− interfaces overlap with the etomidate sites

Recently, both αM236 and βM286 in α1β3 receptors were identified as incorporation sites for a propofol photo-label analog [10]. Our protection studies at α1M236C confirm this locus as a propofol contact residue. Propofol also protects βM286C from thiol modification [13]. Azi-etomidate photolabeling [8] and SCAM protection [12] identify both α1M236 and βM286 as etomidate contact points, and propofol inhibits azi-etomidate photolabeling at these residues [45]. Thus, both αM236 and βM286 are common contact points for both etomidate and propofol. Our results (Figs. 4, 5) also show that βN265M reduces β+/α− site affinity for propofol. Bali & Akabas [13] noted that propofol modulates α1β2N265Cγ2 receptors, and we also observed propofol effects in receptors with βN265M mutations. This likely reflects propofol modulation *via* at least two other transmembrane interfacial sites: β-/α+ and β-/γ+ [45], that also bind a potent barbiturate photolabel [46] but not etomidate. The presence of at least four propofol sites per αβγ GABA_A_ receptor present challenges for interpretation of its effects in mutant channels.

### The βN265M mutation impairs transduction between β+/α− anesthetic sites and channel gating

We further assessed the effect of βN265M on β+/α− anesthetic site transduction by testing the gating effects of α1M236W, a mutation that mimics bound anesthetic [16], [38]. The β2N265M mutation reduced the gating effects of α1M236W mutations about ten-fold, but did not eliminate them (Fig. 6). Extending this conclusion to bound etomidate suggests that reduced efficacy/transduction alone may not fully account for the profound etomidate-insensitivity of receptors containing βN265M mutations. This is consistent with our result from α1M236C protection studies that also show profound effects on anesthetic efficacy combined with more modest, but significant reductions in etomidate affinity for resting state receptors.

The impact of βN265 mutations also appears to be highly selective for β+/α− anesthetic site ligands, further supporting its involvement through local steric interactions. Both GABA EC_50_ and basal channel gating are weakly altered by β2N265S or βN265M mutations [27], and our current results indicate that the same is true for βN265C mutations. Based on our data and others [32], receptors with βN265 mutations also retain modulation by alphaxalone, pentobarbital, and benzodiazepines. Evidence indicates that all of these drugs act primarily *via* allosteric sites that do not overlap with the β+/α− interfacial sites where both etomidate and propofol bind.

### Anesthetics and βN265C protection

In contrast to negative βN265C protection results with etomidate (this study) and propofol [13], McCracken et al [32] reported that n-octanol blocks thiol modification at β2N265C. If all of these anesthetics bind in the β+/α− sites, why don’t they all protect βN265C? One explanation consistent with our results is that βN265C reduces anesthetic affinity more than βN265M; thus β+/α− site occupancy may have been very low during our etomidate protection experiments. Secondly, the possibility that 300 µM etomidate occupies α1β2N265Cγ2L sites without obstructing pCMBS modification cannot be excluded. βN265C forms disulfide bonds with other cysteine substitutions on α-M1, β-M3, and β-M1 [18], revealing potential βN265 interactions with both β+/α− *inter-subunit* anesthetic sites and β *intra-subunit* helix bundles. Structural homology models (e.g. Fig. 1) also show βN265 oriented toward β-M3, lying between intra- and inter-subunit pockets. Other pLGIC subunit helix bundles are known to form sites that anesthetics occupy [47] and the GABA_A_ β helix bundles could be access pathways for pCMBS to reach the thiol of βN265C without interference from propofol or etomidate. If octanol, a small flexible molecule, binds in both β helix bundles and β+/α− sites, this could explain its unique protection at βN265C. Thirdly, the small size and flexibility of n-octanol relative to propofol and etomidate may allow it to bind closer to βN265 within the β+/α− sites. Intriguingly, high-resolution structures of GLIC mutants suggest that the relative size and occupancy of inter-subunit and intra-subunit transmembrane pockets influences whether small alcohols and anesthetics act as gating enhancers *versus* inhibitors [30]. Moreover, the positive modulating anesthetic sites on GLIC are formed between M2 domains on adjacent subunits, rather than between M1 and M3 domains. Thus, examination of anesthetic interactions with both intra-subunit helix bundle pockets and various parts of the inter-subunit pockets of GABA_A_ receptor may reveal distinct sites where small versus large drugs bind and act.

### βN265 and etomidate photolabels

If βN265 sidechains contact anesthetics, how also to explain the absence of anesthetic photolabeling at this residue? The large effects of βN265 mutations on etomidate sensitivity are fully consistent with complementary structure-function data on etomidate derivatives, if we assume limitations on their binding orientation. Modification at the ester leaving group of etomidate, including appending large photoreactive groups, preserves anesthetic activity and GABA_A_ receptor modulating efficacy [48], [49]. Photolabels of this type label residues on α–M1 and β-M3, near the lipid-protein interface. In contrast, based on inhibition of azi-etomidate labeling, p-trifluorodiaziryl (TFD) substitution on the phenyl ring of etomidate reduces affinity for etomidate sites ∼50-fold relative to a similar p-TFD-benzyl substitution at the ester position [45]. Other bulky additions to the phenylethyl group or reorientation around the chiral carbon adjacent to the phenyl group of etomidate also dramatically reduce anesthetic activity in animals and/or modulatory efficacy in GABA_A_ receptors [49]–[53]. Together, these results suggest that the phenylethyl end of bound etomidate is oriented toward the transmembrane pore and M2 domains (i.e. βN265), in a sterically constrained posture that likely contributes to etomidate stereoselectivity.

Finally, GABA_A_ receptors form other transmembrane anesthetic sites, including other inter-subunit pockets, intra-subunit helical bundles, and the ion channel [54], [55]. Different anesthetics display distinctive specificities for these various sites [10], [45]. Defining the pLGIC structural elements involved in site-selective anesthetic binding will be essential to understanding the mechanisms of these important drugs, while also informing development of more receptor sub-type selective modulators.

## Supporting Information

Figure S1 **Alignment of GABA_A_ receptor subunit amino acid sequences with GluCl.** Each subunit sequence was independently aligned as described in methods. Predicted secondary structure domains are indicated by blue bars for beta sheet and red bars for alpha-helix.
(TIF)
