# Supplementary figures and images for: Mutations at Beta N265 in γ-Aminobutyric Acid Type A Receptors Alter Both Binding Affinity and Efficacy of Potent Anesthetics
Source: PLoS One. 2014 Oct 27;9(10):e111470. doi: 10.1371/journal.pone.0111470 (PMC4210246; doi:10.1371/journal.pone.0111470)

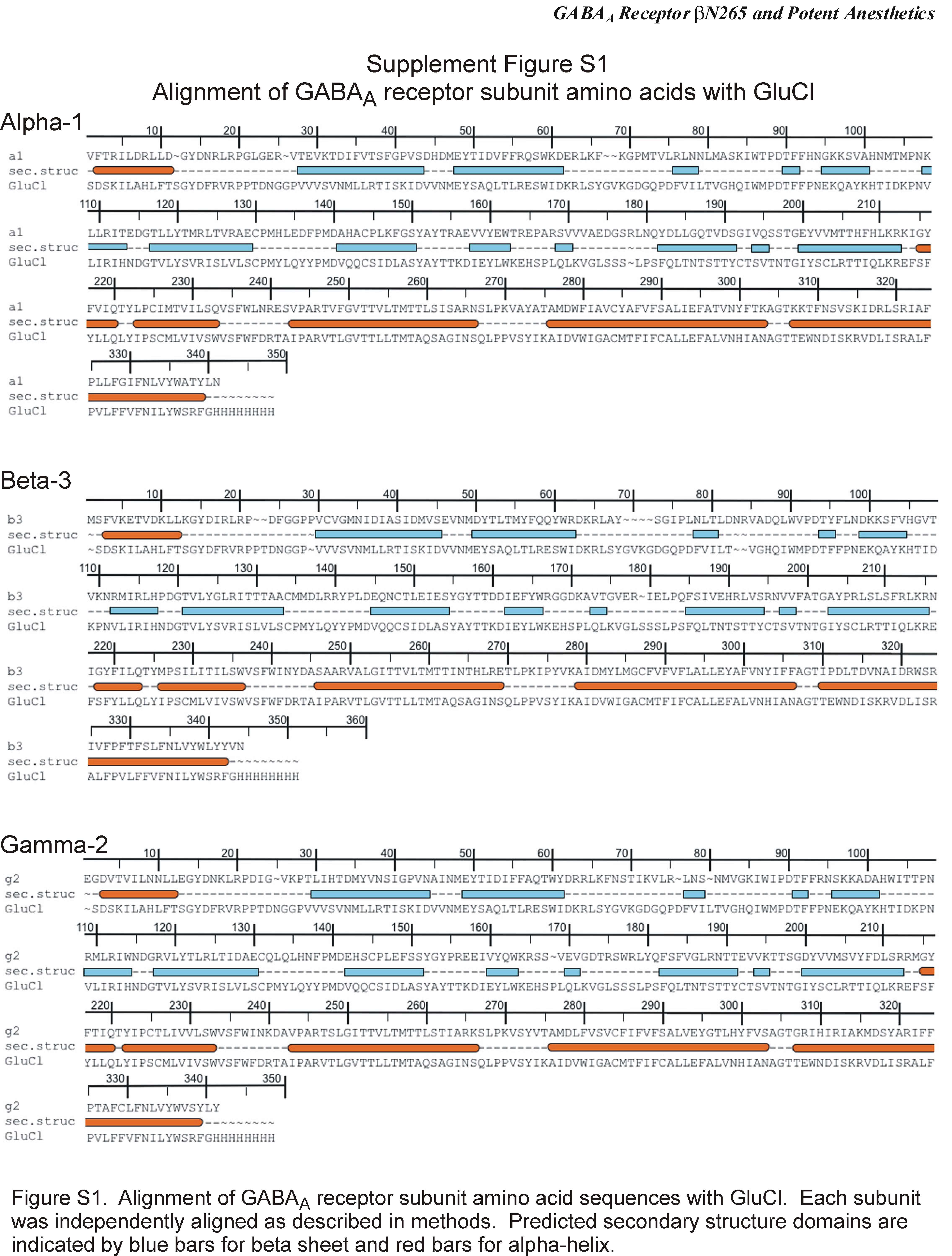

Supplement: Figure S1 — Alignment of GABAA receptor subunit amino acid sequences with GluCl. Each subunit sequence was independently aligned as described in methods. Predicted secondary structure domains are indicated by blue bars for beta sheet and red bars for alpha-helix. (TIF) [file pone.0111470.s001.tif]
